# The 3-Omega Method for the Measurement of Fouling Thickness, the Liquid Flow Rate, and Surface Contact

**DOI:** 10.3390/s17030552

**Published:** 2017-03-09

**Authors:** Casper Clausen, Tim Pedersen, Anders Bentien

**Affiliations:** Department of Engineering, Aarhus University, Hangoevej 2, 8200 Aarhus N, Denmark; casperc@eng.au.dk (C.C); tim.pedersen@post.au.dk (T.P.)

**Keywords:** sensors, 3-omega, biofouling, fouling, flow, contact

## Abstract

The 3-omega method is conventionally used for the measurement of thermal conductivity in solid samples. The present work includes the experimental characterization and proof-of-concept measurements of sensor concepts, based on the 3-omega method. It is shown that this method can be used to measure fouling layers with a thickness of 10 to 400 µm, to conduct the measurement of flow rates with a high precision, and finally, as a simple on-off contact sensor with a fast response time.

## 1. Introduction

The 3-omega (3*ω*) method [1,2,3,4,5] is a well-established technique for measuring the thermal conductivity (κ) of solid samples, particularly for samples with low thermal conductivity [1]. In modified configurations, it has also been applied to the measurement of the thermal conductivity of gases [6,7] and fluids [8,9], also including fluids in flow [9]. For the past decade, the 3*ω* method has been investigated with respect to the measurement of flow speeds [10,11], liquid levels [12], and has been used as a sensor for the measurement of cell viability [13]. 

**Figure 1:**
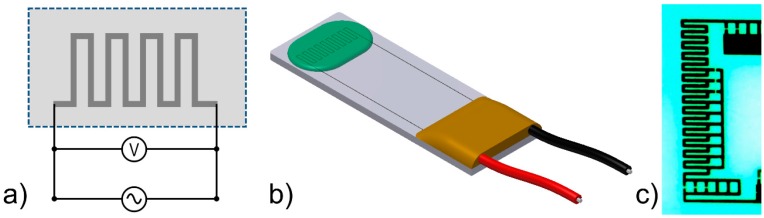
(**a**) Schematic outline of the electrical circuit used in the experiments; (**b**) Simplified illustration of the IST FS5 sensor showing the rectangular ceramic substrate with the platinum thin film on top. The green part illustrates a layer of e.g., biofouling; (**c**) Magnified picture of the sensor tip.

The basic electronic circuit for the 3*ω* method is schematically shown in Figure 1a. An AC current (I) with an angular frequency (*ω*), $I\left(t\right)=I_{0}\;\sin\left(\omega t\right)$, passes through a thin metal film deposited on a substrate; the dimensions of which are provided by the width (*w*), thickness (*h*), and length (*l*) values. Due to Joule heating, the temperature of the thin film and the surrounding oscillates have a frequency that is twice (2*ω*) that of the base frequency. Because of the temperature coefficient of resistance (α) for the metal film, the resistance (*R*) of the metal film also oscillates with 2*ω*. The measured electrical potential difference across the film (Figure 1a) includes a complex 3*ω* component ($U_{3\omega }$) that arises from the product between the 2*ω* and 1*ω* oscillating resistance and current, respectively. 

In order to establish analytical expressions for the relations between $U_{3\omega }$ and κ, it is necessary to impose geometrical limitations on the thin film. i.e., if the width of the thin film is much smaller than the thermal wavelength or penetration depth ($\lambda =\sqrt{D/2\omega }$ where D is the thermal diffusitivity of the surroundings of the thin film), the magnitude of temperature oscillations of the thin film is given by [1]:

(1) $$\Delta T=\frac{2}{\alpha }\cdot \frac{U_{3\omega }}{U_{1\omega }}$$

(2) $$\Delta T=\frac{P}{l\pi \kappa }\left(-\frac{1}{2}\ln\omega +k\right)$$

where $U_{1\omega }$ is the magnitude of the 1*ω* component, P is the electrical Joule heating dissipated in the metal film, and k is a frequency independent complex constant that depends on the thermal surroundings and geometry of the thin film [2]. The 3*ω* method, used for probing the thermal properties of the surroundings, can also be applied to dimensions other than those which are mentioned by Cahill, and this has been studied both experimentally and theoretically [6,14]. In all cases, the general trend is that $U_{3\omega }$ decreases with an increasing thermal conductivity of the surroundings (the substrate and media above the metal film). This effect can be used for a range of applications other than the measurement of the thermal conductivity, and in the present study, the 3*ω* method is experimentally investigated for applications within fouling sensing, flow sensing in liquids, and on-off contact sensing.

## 2. Experimental

The sensor used for the experiments is produced by Innovative Sensor Technology (IST FS5.0.1L.195, Ebnat-Kappel, Switzerland) and is illustrated in Figure 1b. With dimensions of 6.9 × 2.4 × 0.2 mm^3^, it consists of a ceramic substrate with a low thermal conductivity, a platinum resistor in a serpentine pattern, and a thin coating of about a 30 µm thickness for protection of the metal film. The material used for the protective layer is unknown to the authors, but since it is present on the sensor in all measurements, the inevitable effect which the layer has on the measurement is therefore considered as a constant offset, equal for all results. The sensor is manufactured for thermal gas flow sensing applications and has two different platinum thin films, but in the present study, only one was used. The electrical circuit is outlined in Figure 1a, while Figure 1b is an illustration of the sensor. Figure 1c is a magnified picture of the sensor tip showing the thin film circuit, where the width of the thin film is approximately 30 µm, and the total active length is approximately 8.65 mm. The AC current is supplied by a Keithley Instruments (Cleveland, OH, USA) 6221 AC and DC current source, and the voltage signal is measured with a 16 bit National Instruments (Austin, Texas, USA) USB-6210 ADC in a four-wire set-up. Data logging and analysis are made with LabVIEW (National Instruments, Austin, TX, USA), and in all experiments, the sampling frequency is 10 kHz, unless otherwise stated. 

## 3. Sensor Characterization and Data Analysis

**Figure 2:**
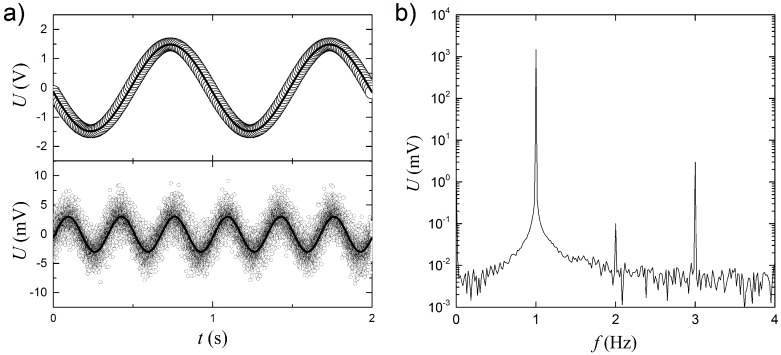
(**a**) Example of raw voltage signal as function of time ($U_{\mathrm{raw}}\left(t\right)$). The upper panel shows the raw data fitted with a sinusoidal function (solid line). Only every 100th data point is shown. The lower panel shows $U_{3\omega }\left(t\right)=U_{\mathrm{raw}}\left(t\right)-U_{1\omega ,\mathrm{fitted}}\left(t\right)$ of the same set of data with all data points shown and a new fit to a sinusoidal function (solid line); (**b**) Amplitude of the Fourier transform of the raw data in (**a**) over the complete 60 s time series.

For the characterization of the initial sensor and the determination of optimal currents and frequencies, the sensor characteristics were measured in liquid water at room temperature (23 °C). The upper panel of Figure 2a shows an example of the raw voltage ($U_{\mathrm{raw}}\left(t\right)$) signal, sampled in non-flowing water with a base frequency of 1 Hz. For demonstrating the raw $U_{3\omega }\left(t\right)$ signal in the time domain, a sinusoidal fit of $U_{1\omega }\left(t\right)$ with a frequency of 1ω is subtracted from $U_{\mathrm{raw}}\left(t\right)$, and the result is shown in the lower panel of Figure 2a. It is clearly seen that the obtained signal oscillates with a frequency of 3ω. After a new sinusoidal fit of these data, the amplitude of $U_{3\omega }$ is found to be 3.0 mV, which is about three orders of magnitude lower than the $U_{1\omega }=$ 1.48 V amplitude.

Fourier transforms are used to extract the magnitude of $U_{3\omega }$ throughout this paper. As Figure 2b shows, the Fourier transform of the raw data in the upper panel of Figure 2a, the whole time series of 60 s has been included. It is seen that there is a good signal to noise ratio of more than 100 for the $U_{3\omega }$ signal. The amplitudes are found to be $U_{1\omega }=$ 1.49 V and $U_{3\omega }=$ 3.0 mV, respectively, and are in very good agreement with the results from the sinusoidal fits in the raw data analysis. Furthermore, it is noted that the noise level is <0.01 mV and lower than the formal resolution of ~0.076 mV of the 16 bit ADC, because the time series are measured over 60 s, thus improving the practical resolution beyond 16 bit. Unless otherwise stated, the $U_{3\omega }$ amplitudes are obtained from the Fourier transforms of raw data over at least 10 AC cycles and phase information is not used in the data analysis. 

**Figure 3:**
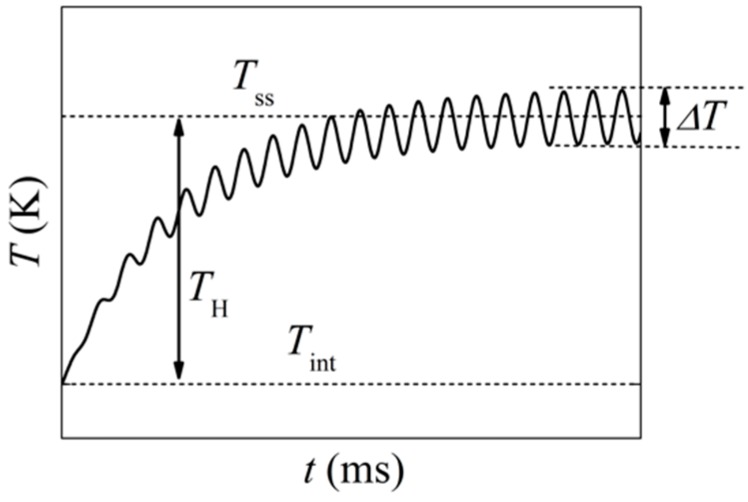
Illustration of the temperature of the metal film as a function of time after starting the measurement. The total time dependence of the temperature is a sum of the self-heating ($T_{H}$) and an oscillating temperature with a frequency of 2ω.

The choice of the magnitude of the AC current ($I_{0}$) is a trade-off between a high $U_{3\omega }$ signal and a low temperature increase, due to the self-heating ($T_{H}$) of the sensor that increases with $I_{0}^{2}$, due to Joule heating. Figure 3 shows the general development of the temperature over time. At the beginning of the experiment, the temperature of the sensor increases, until a steady-state is reached after less than 200 ms. Then, the sensor dissipates heat to the surroundings with the same average rate as is produced from Joule heating. 

$T_{H}$ can be estimated from:

(3) $$T_{H}\left(I\right)=\frac{T_{\mathrm{int}}}{R_{\mathrm{int}}}\cdot R_{H}\left(I\right)=T_{\mathrm{int}}\left(\frac{R_{\mathrm{ss}}\left(I\right)}{R_{\mathrm{int}}}-1\right)$$

where $T_{\mathrm{int}}$ is the initial absolute temperature; $R_{\mathrm{int}}$ is the initial electrical resistance of the platinum thin film at temperature $T_{\mathrm{int}}$; and $R_{H}$ is the change that can be seen once the self-heating has reached a steady state, at which point the electrical resistance becomes $R_{\mathrm{ss}}=R_{\mathrm{int}}+R_{H}$. This approximation holds for metals with positive temperature coefficients and in situations when the resistance is a linear function of the temperature, as can be seen for platinum above the low temperature resistance plateau (~50 K). The resistance can be calculated from the measured $U_{1\omega }$ amplitude and the current amplitude $I_{0}$.

**Figure 4:**
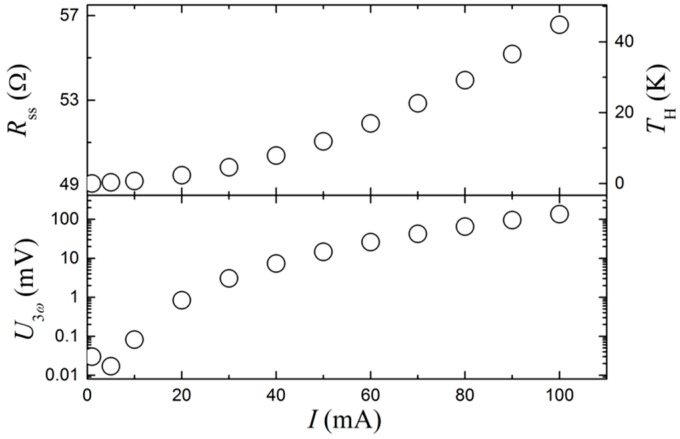
The upper panel shows the electrical resistance (R) and self-heating temperature ($T_{H}$) as a function of the current. The lower panel shows the amplitude of the 3ω signal ($U_{3\omega }$) as a function of the current.

Figure 4 shows the result of a self-heating experiment, where the frequency is fixed at 1 Hz and without flow. The upper panel shows $R_{\mathrm{ss}}$ and $T_{H}$, while the lower panel shows $U_{3\omega }$ as a function of the applied current on a logarithmic scale. An approximate self-heating of 3 K (1% of the absolute temperature) is chosen as an acceptable limit, corresponding to a current of 30 mA. This is a trade-off between a large $U_{3\omega }$ signal and a low self-heating value that can potentially interfere with the measurement. With this current, $U_{3\omega }=$ 3.1 mV and is several orders of magnitude above the detection limit of the ADC. Unless otherwise stated, a current amplitude of 30 mA is used in the following experiments.

**Figure 5:**
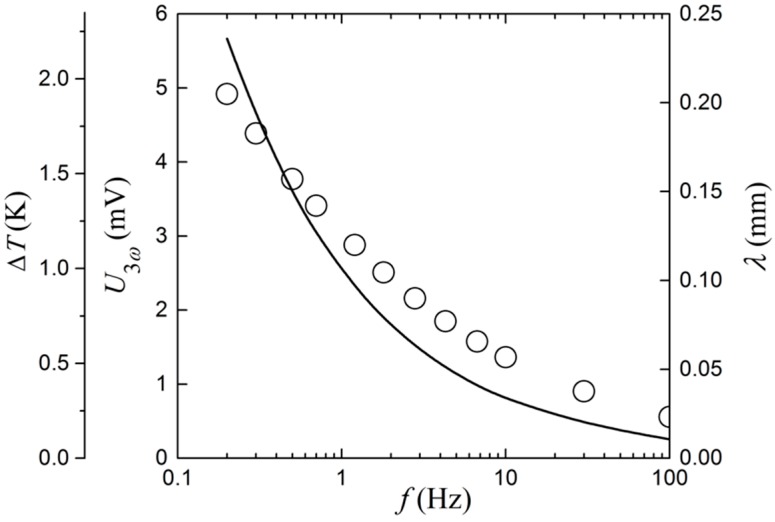
Amplitude of the 3ω signal, and temperature oscillations (left y-axes, circles) and thermal wavelength (right y-axis, continuous line) as functions of the frequency.

Figure 5 shows how the amplitude of the 3ω signal depends on the frequency without flow around the sensor. At frequencies below approximately 1 Hz, $U_{3\omega }$ is a linear function of logf, as expected in the low frequency limit [1]. Figure 5 also includes the magnitude of the temperature oscillations (ΔT), assuming that the Cahill formula [2] for $\Delta T=2U_{3\omega }/\left(\alpha U_{1\omega }\right)$ holds. Here, it is seen that the maximum ΔT is less than 2 K at 0.2 Hz. Additionally, it presents the thermal wavelength $\lambda =\sqrt{D/2\omega }$, where the thermal diffusivity for water (D= 0.145 mm^2^/s^−1^) has been used [15]. λ decreases from about 0.24 mm at 0.2 Hz, to only 0.01 mm at 100 Hz. The thermal wavelength or thermal penetration depth is a rough measure of the maximum measurement depth of e.g., the fouling thickness. When choosing the driving frequency for a measurement, there is a trade-off between a large signal amplitude ($U_{3\omega }$) with a large measurement depth on the one hand, and a fast response time on the other. A minimum measurement of 10 cycles is kept as a criterion, and unless otherwise stated, a frequency of 1 Hz is used throughout the experiments in this work. When conducted in water at room temperature, this results in $U_{3\omega }\sim$3 mV, a thermal penetration depth of about 120 µm, and a minimum measurement time of 10 s.

### Temperature Invariance

**Figure 6:**
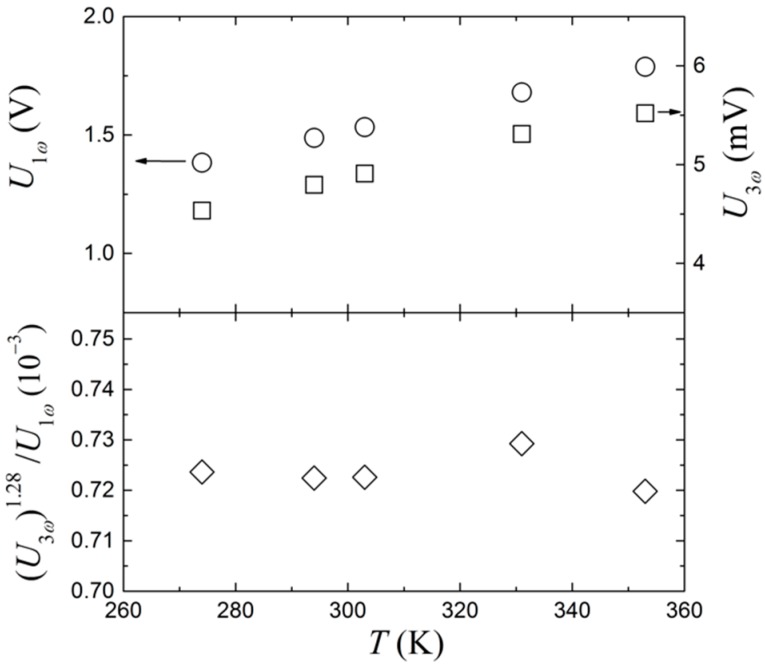
The upper graph shows $U_{1\omega }$ and $U_{3\omega }$ as functions of temperature, without stirring. The measurements are all completed with an AC current of 30 mA and a driving frequency of 0.2 Hz. The lower graph shows the ratios between $U_{3\omega }$ and $U_{1\omega }$ from the upper graph. Here, $U_{3\omega }$ is raised to the power of *n* = 1.28, to correct for the nonlinear behavior at higher temperatures.

Varying temperatures can be a challenge in resistance-based sensors, where small changes in temperature can have a significant influence on the sensor response. Since both $U_{1\omega }$ and $U_{3\omega }$ are influenced by the resistance change of the thin film, it appears feasible to investigate the ratio between the amplitudes of these two signals. Both signals are recorded at different temperatures, in the range of 274 to 353 K. The driving frequency is varied from 0.2 to 100 Hz, but the initial analysis is made with the 0.2 Hz data since these give the largest signals and are thus more sensitive to temperature variations. The data can be seen in Figure 6, where $U_{1\omega }$ linearly increases with temperature, as expected for a metal like platinum. However, $U_{3\omega }$ increases with the temperature with a slightly lower power, as $U_{1\omega }$ and a simple ratio between the two will not be temperature independent. To correct for this behavior, $U_{3\omega }$ is raised to the power of n, where a value of 1.28 has been empirically found to produce the best result (see lower graph in Figure 6), at which point it becomes fully temperature independent within the experimental uncertainties.

**Figure 7:**
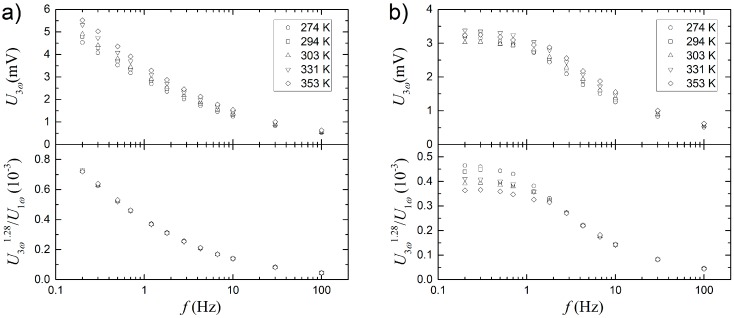
Frequency dependence of $U_{1\omega }$ and $U_{3\omega \;}$ at different temperatures. The top panels show the raw measurements of $U_{3\omega }$ and the bottom panels show ${\left(U_{3\omega }\right)}^{1.28}/U_{1\omega }$. (**a**) Stirring is off; (**b**) Stirring is on.

The same result is obtained for measurements at other frequencies and is shown in Figure 7a, where $U_{3\omega }$ is plotted as a function of frequency for different temperatures. The top panel shows the uncorrected $U_{3\omega }$ at different temperatures and frequencies, while the bottom panel shows the same data, but uses ${\left(U_{3\omega }\right)}^{1.28}/U_{1\omega }$ as a function of frequency. Again, it is seen that it becomes temperature independent over the whole frequency range. To investigate the effect of flow around the sensor, Figure 7b shows a similar measurement. However, this time it is measured in a stirred beaker. Using the same value for *n* does not result in temperature invariance in the low frequency range. 

We believe that the physical explanation of the magnitude of n is a consequence of the temperature dependence of the substrate and/or water. From Equation (2), it is seen that ΔT∝P/κ, where $P=RI_{0}^{2}$ is the Joule heating, and combined with Equation (1), it can be found that $U_{3\omega }/U_{1\omega }\propto \alpha RI_{0}^{2}/\kappa$. From good approximations, it can be assumed that $\alpha \propto T^{-1}$ and R∝T, whereby it is seen that $U_{3\omega }/U_{1\omega }\propto \kappa^{-1}$. If κ is temperature independent, $U_{3\omega }/U_{1\omega }$ becomes constant, and we assess that the departure from the n = 1 relationship is a consequence of the temperature dependence of κ on the surroundings of the metal film.

## 4. Results and Discussion of Application Tests

### 4.1. Fouling Sensor Applications

**Figure 8:**
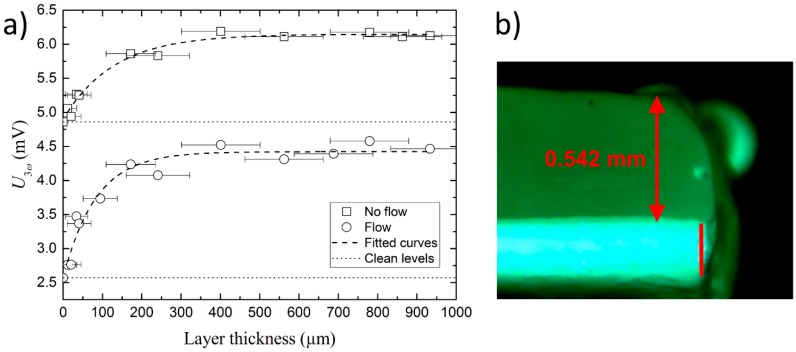
(**a**) $U_{3\omega }$ as a function of biofilm layer thickness. The data follows a reverse exponential decay function within the measured range, as shown with the dashed lines. The dotted lines show the signal levels for the clean sensor; (**b**) Microscope side view image of the sensor with a layer of wax.

For initial tests as fouling sensor applications, a layer with a well-known thickness is artificially applied to the sensor. A range of substances are tested and it is found that non-water-based hair wax presents the most advantages in terms of depositing layers with well-defined thicknesses and robustness. The wax is applied using a digital micrometer screw to control the thickness of the layer. The actual layer thickness and smoothness is then measured under a microscope (see Figure 8b). The sensor with the deposited layer is placed in a beaker with water and the voltage signal is measured using a driving frequency of 0.2 Hz and an AC current of 30 mA. The signal is measured both with and without stirring in the beaker, to simulate the effects from flow around the sensor. This procedure is repeated for different layer thicknesses, ranging from 11 to 933 µm, and the result can be seen in Figure 8. For both the experiment with and without flow, a clear monotonic increase of $U_{3\omega }$ as a function of the layer thickness is seen up to about 200–400 µm, after which the response flattens out. The dashed line is a least squares fit to the data, with a reverse exponential decay function. It is seen that there is a relatively large deviation for some points from the fit, and it is anticipated that this deviation is not due to the measurement uncertainty of $U_{3\omega }$, but is related to the measurement uncertainty of the actual wax layer thickness. In particular, it was difficult to create perfectly uniform layers around the sensor edge where the metal film is situated and the uncertainty has been estimated to be 25% of the layer thickness, but with a minimum and maximum of 20 µm and 100 µm, respectively.

Nonetheless, it is clear that the response and sensitivity of the sensor are highest for thicknesses below 200 µm, while above this value, they are much lower. 

A response of >200 µm is related to the thermal penetration depth of the $U_{3\omega \;}$ wave $\lambda =\sqrt{D/2\omega }\sim$ 200 µm, estimated from D= 0.098 mm^2^/s for paraffin [16], which is similar to the lipids which are the main constituent of hair wax. Above this thickness, the thermal oscillations are diminished and no longer contribute to the sensor response. Nonetheless, higher λ and maximum measurement thicknesses can be obtained if a lower driving frequency is used.

Comparing the tests with and without flow around the sensor, it is seen that the response of the sensor is lower with flow, compared to without flow. Furthermore it is also seen that the offset without flow is larger. Together, this means that a sensor has a higher sensitivity with flow. The reason for this is that flow around the sensor effectively corresponds to surroundings with larger thermal conductivity, because of convection. Whenever a thermally isolating layer is deposited, this will result in a larger response in the sensor. 

**Figure 9:**
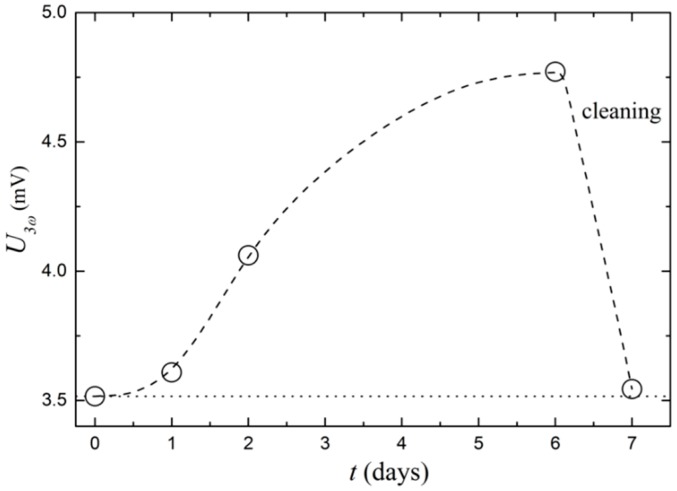
Sensor in a bacterial growth environment showing $U_{3\omega }$ as a function of time over six days. On day seven, the sensor is cleaned and $U_{3\omega }$ regains its initial value. The dashed line is a guide to the eye and the dotted line is the $U_{3\omega }$ magnitude for the clean sensor at day zero.

To further test the measurement of fouling layers, the sensor is exposed to an environment prone to developing biofilm over time. This is made from a colony of *Staphylococcus xylosus* in a 1% weight concentration of the growth medium Tryptic Soy Broth, in distilled water. The sensor is placed in the bacterial growth environment with stirring and $U_{3\omega \;}$ is measured at day zero. Subsequent measurements are taken after one, two, and six days. On day seven, the sensor is cleaned with ethanol and $U_{3\omega \;}$ is measured again, to test the sensor’s ability to reach the initial value. As seen in Figure 9, $U_{3\omega \;}$ increases during the bacterial growth period and returns to the initial value after cleaning. We attempted to measure the biofilm thickness in a microscope, but this was unsuccessful, because the layer is transparent and is estimated to be less than 100 µm. Nonetheless, the fact that cleaning the sensor surface brings it back to the initial state, clearly demonstrates the ability to measure even very thin layers of biofilm.

Compared to state-of-art products, there exists, to the authors’ knowledge, only one commercially available technology for the measurement of fouling and bio-films (FS-1000 from the company NeoSens (Champagny-sur-Marne, France) [17]), and it is based on a thermal DC technique. The general advantages of AC compared to DC measurement techniques are an increased sensitivity and faster measurement times, because data filtering is inherent to the AC measurement and a steady-state is reached within a few cycles of the AC measurement frequency, respectively. 

### 4.2. Flow Sensor Applications

In state-of-art thermal mass flow meters, the current is applied to a metal film (heater). Depending on the flow speed and flow direction, this heats up the flowing medium and can be measured as an increased temperature in a second metal film (temperature sensor), which is in the close vicinity of the heater. The main difference from the conventional thermal mass flow principle and the 3*ω* method is the use of only one metal film, which acts as both the excitation and the measurement metal film at frequencies 1*ω* and 3*ω*, respectively. As mentioned earlier, the advantages of AC measurement techniques include faster measurement times, because a steady-state is reached faster, and an increased sensitivity, because data filtering is inherent to the AC measurement. 

Using the 3*ω* method to measure flow speed has previously been investigated [10,11]. Ref. [10] uses a wire as an alternative to a thin film; however, this leads to a complicated data analysis that involves the frequency dependence of the 3*ω* signal. The method in ref. [11] is based on a thin film and measures the flow speed from the phase angle of the 3ω signal, which only demonstrates a small temperature dependence. In the present study, the magnitude of the 3*ω* signal is used for the determination of the flow speed. In the experimental setup, the sensor is placed inside a 3 mm narrow silicon tube, which is made by pushing the sensor through a small cut in the tube wall. The sensor is placed far from the pump, to ensure a fully developed flow. Water is circulated through the tube using a 5–12 V submersible water pump, controlled by an AIM-TTI EL302R DC power supply (Aim-TTi, Huntingdon, UK).

**Figure 10:**
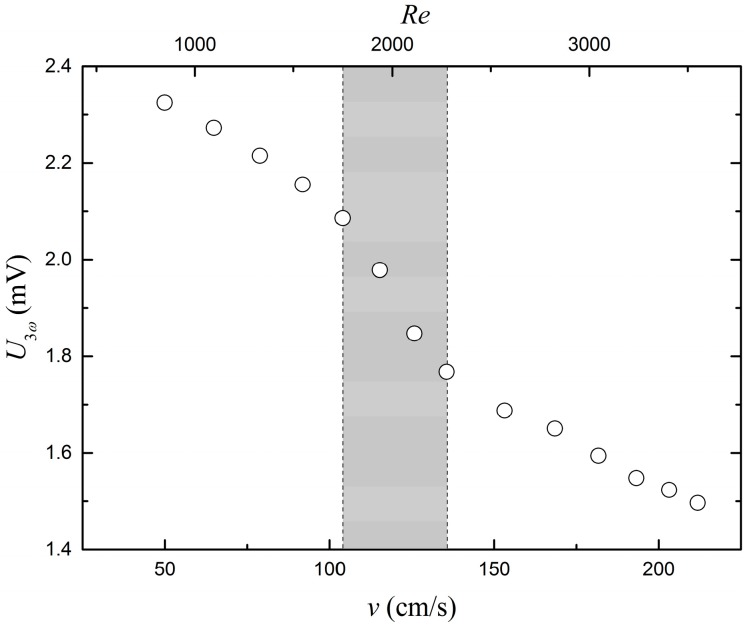
Amplitude of the 3ω signal as a function of the linear flow speed of the water in contact with the sensor. The upper *x*-axis shows the Reynolds numbers corresponding to the flow speeds. The standard deviations of the data are smaller than the data points on the graph.

**Figure 11:**
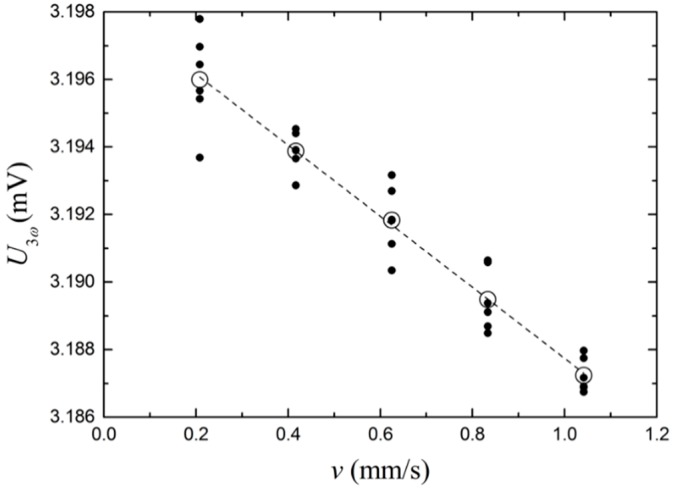
Amplitude and standard deviations of the 3ω signal as a function of the linear flow speed of the water in contact with the sensor. A least squares linear fit is shown with a dashed line.

Figure 10 shows the $U_{3\omega }$ response at different flow rates. A shift in the generally linear trend appears just above 1 m/s, which corresponds to a Reynolds number of about 2000, which is the normal onset for laminar to turbulent flow. In this range, marked by the grey section in Figure 11, a larger decrease in $U_{3\omega }$ is observed. Once the flow reaches the fully turbulent flow $U_{3\omega }$ it follows the same slope as when the flow is laminar. The functional behavior in Figure 10 is fully in agreement with that which was expected. An increased flow speed transports heat away more efficiently, corresponding to a larger thermal conductivity and lower $U_{3\omega }$. It is also well-known that the heat transfer coefficient for turbulent flow is higher than for laminar flow, and for this reason, the slope in the flow transition range is higher. 

Figure 11 shows $U_{3\omega }$ as a function of the flow speed at much lower flow velocities, obtained by the combined use of a flow cell with a larger cross sectional area and a WPI AL-1000 (World Precision Instruments, Sarasota, FL, USA) syringe pump for low flow rates. Here, data are collected over a period of 60 s, and the data for different succeeding experiments with the same flow speed are plotted as solid data points. At the same time, the average values for the five data points are included as open data points. The dashed line is a least squares linear fit to the data, where the slope is 0.0105 V/(m/s). From the variance of the data points between each flow speed, the sensitivity is estimated to be around 0.2 mm/s for measurements recorded over 60 s, but much lower if data are averaged over several data points, as seen from the coincidence of the fitted curve and the averaged data points. This demonstrates one of the advantages of AC techniques and the ability of low flow speed measurements. 

Conclusively, the sensor is able to measure different flow rates and has a high dynamic range of >5000. Furthermore, the sensor also has the ability to identify the shift from laminar to turbulent flow, which can be useful when handling liquids with an unknown viscosity and/or density.

### 4.3. Contact Sensor Applications

**Figure 12:**
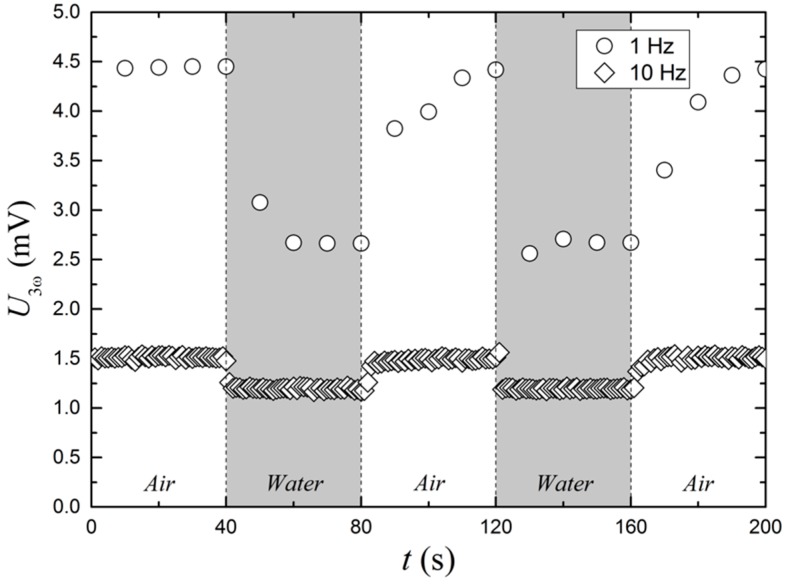
The graph shows the sensors response to alternately being surrounded by air and water. Two different datasets with different driving frequencies are presented. The temperature of the water and the air is the same.

With a factor of more than 20 between the thermal conductivities of water (606.2 mW/(m·K)) and air (26.2 mW/(m·K)) [15], it is obvious that a sensor utilizing a thermal conductivity measurement like the 3*ω* method can be used as a contact sensor for the measurement of the presence of water or other liquids and objects touching the sensor. Here, one of the main issues is the response time of the sensor. A minimum of 10 AC cycles is demanded for an acceptable quality of the Fourier transform and a measurement time of 10 s is needed for a driving AC frequency of 1 Hz. The result of such a measurement can be seen in Figure 12, where $U_{3\omega \;}$ amplitudes of 4.45 and 2.66 mV for air and water, respectively, are observed. To decrease the response time, the driving frequency is increased to 10 Hz and the measurement time reduced to 1 s. The improvement of the response time comes with the cost of the reduced $U_{3\omega }$ amplitude, i.e., 1.51 and 1.19 mV for air and water, respectively. The ratio of the signal in air to the signal in water is also reduced from 1.67 to 1.26, when changing the driving frequency from 1 to 10 Hz.

Compared to state-of-art contact sensors, the advantages of the 3*ω* sensor are its small footprint and the absence of fragile mechanical parts.

## 5. Summary and Conclusions

Various new uses of the 3*ω* method have successfully been demonstrated. The method is conventionally used for the thermal conductivity determination of different materials, but in this study, it has been shown to contain a much more diverse range of possibilities. All results are found using a small gas flow sensor from Innovative Sensor Technology (IST), driven by an AC current source.

For biofilm detection, it is shown that biofouling thicknesses between 10 and 400 µm, made with non-water based hair wax, result in 3ω signal amplitudes between 2.7 and 4.6 mV. The correlation follows a reverse exponential decay function with a correlation coefficient above 0.936. The sensor signal is able to return to the baseline after cleaning.

For low water flow rate measurements, a 3ω signal amplitude range between 3.196 and 3.187 mV is the result of linear water flow rates between 0.208 and 1.04 mm/s. A linear correlation with R-squared >0.999 is found within this range. For higher flow rates, the method is able to detect the transition between laminar and turbulent flow. The signal follows a linear trend as a function of the flow rate in both regions with a laminar flow, producing larger signals than turbulent flows.

Using the 3*ω* method with the IST sensor to distinguish between being in direct contact with air and water, allows the technology to be used as a water level sensor. The response time and signal amplitude are mutually dependent, so that shorter response times produce lower signal amplitudes. In this work, a response time of 1 s has been shown to give an air-to-water signal amplitude ratio of 1.26.

The advantages of the method are mainly related to the simplicity of both the design and the supporting electronics having no mechanical parts and only one resistor acting simultaneously as excitator and detector, the low-cost potential, and the possibility to simultaneously use it as a thermometer with the desired application. 

## Abbreviations

The following abbreviations are used in this manuscript:

IST	Innovative Sensor Technology
ADC	Analog-Digital Converter & Data Acquisition
AC	Alternating Current
DC	Direct Current
